# Almost there: learning to navigate approximately with a grid map

**DOI:** 10.1016/j.beproc.2025.105245

**Published:** 2025-08-05

**Authors:** Joe Morford, Patrick Lewin, Paris Jaggers, Joe Wynn, Tim Guilford, Oliver Padget

**Affiliations:** 1 Department of Biology, University of Oxford, Oxford, UK; 2 University College London, London, UK; 3 Institut für Vogelforschung “Vogelwarte Helgoland”, Wilhelmshaven, Germany; 4 Department of Earth, Ocean and Ecological Sciences, University of Liverpool, Liverpool, UK

**Keywords:** Grid map navigation, environmental gradients, the approximate model, artificial neural networks

## Abstract

Grid map navigation, in which animals use intersecting environmental gradients to judge the spatial relationship between their location and their goal, has been proposed to account for impressive navigational abilities across various taxa. However, the precise mechanisms by which animals navigate using environmental gradients are obscure: first, how do animals extrapolate the spatial distribution of gradients, and second, how do they combine spatial information from multiple gradients? Various models of the extrapolation and combination of spatial gradients have been proposed, but the ontogeny of these mechanisms is little considered. Animals might be predisposed to utilise particular navigational strategies, with these fixed through development; alternatively, mechanisms might arise and change through learning. To investigate this, we trained artificial neural networks, as simple computational learning models, to navigate in virtual bicoordinate grid environments, and tested their outputs against previously proposed models. We found neural networks initially adopted ‘the approximate model’: determining their displacement in each gradient independently and summing these to approximate goalward directions. This supports the suggestion that this model represents a relatively simple mechanism to adopt in complex environments. However, by the end of training, neural networks no longer conformed to the model predictions, hence adopting this mechanism for a limited period only. Thus, the predictions of these models might be met only in certain developmental stages as animals learn. Conversely, the neural networks extrapolated gradients differently depending on the environment. These results facilitate more nuanced predictions of how animal navigation might develop through learning. These predictions should be tested as large tracking datasets of animal movements accumulate.

## Introduction

The idea that animals navigate with a grid map of intersecting environmental gradients has been repeatedly suggested to account for impressive navigation abilities across a range of taxa (Baker, 1984; Gould, 1982; Papi, 1990; Putman et al., 2011; Wallraff, 1974; Wallraff, 2004; Yeagley & Whitmore, 1947). In particular, grid maps have been invoked to explain observations of true navigation, the ability to navigate to a goal from novel, far-off sites, without access to familiar landmarks, goal-emanating cues, or information about the displacement route (Griffin, 1952; Holland, 2014; Keeton, 1974). Grid map navigation is a type of map-and compass navigation, involving two distinct steps: determining direction (and potentially distance) to a goal using a ‘map’, and aligning this to the current egocentric heading using a ‘compass’ in order to orient towards the goal (Kramer, 1953). Navigating with a grid map exploits the continuous and predictable distribution of perceptible gradients in space, facilitating goalward navigation flexibly through space. Various components of the magnetic field are distributed along spatial gradients and appear to be utilised by animals as map cues (Benhamou et al., 2011; Chernetsov et al., 2008; Chernetsov et al., 2017; Kishkinev et al., 2020; Kishkinev et al., 2013; Kishkinev et al., 2010; Kishkinev et al., 2015; Kishkinev et al., 2021; Lohmann & Lohmann, 1994; Lohmann et al., 2001; Lohmann & Lohmann, 1996; Lohmann et al., 2004; Luschi et al., 2007; Minkoff et al., 2020; Pakhomov et al., 2018; Putman, 2015; Putman et al., 2011; Putman et al., 2013; Putman et al., 2014; Putman et al., 2015; Putman et al., 2020; Scanlan et al., 2018). Similarly, some animals have been shown to utilise an olfactory map in their long-distance navigation, including homing pigeons (for reviews, see (Gagliardo, 2013; Wallraff, 2004)), and seabirds (Benvenuti et al., 1993; Gagliardo et al., 2013; Padget et al., 2017; Pollonara et al., 2015), potentially facilitated by stable spatial gradients of olfactory cues over large spatial scales (Wallraff, 2000; Wallraff, 2013; Wallraff & Andreae, 2000; Zannoni et al., 2020).

**Figure 1:**
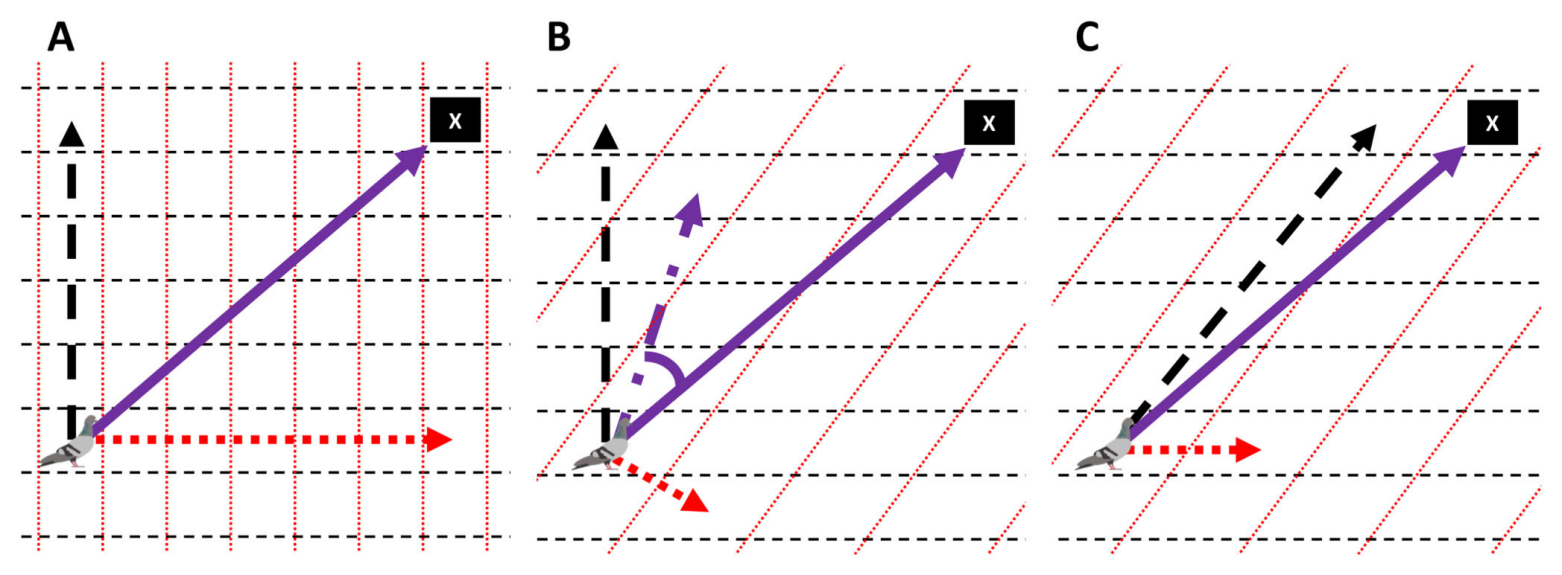
Navigating by vector addition, as in Benhamou, 2003, in orthogonal and non-orthogonal grids. An animal is shown navigating towards a goal using two gradients (in black dashed and red dotted lines). In panels A and B, the animal is shown computing its displacement in each gradient field independently (the black dashed and red dotted arrows) and then using vector summation to determine an overall vector of displacement from its goal. In an orthogonal grid (A), this produces an exact vector towards the goal (purple solid arrow); in a non-orthogonal grid (B), this produces an approximate vector (purple dot-dash arrow) towards the goal, with a predictable error in orientation (shown marked between purple dot-dash arrow and purple solid arrow). Panel C shows how taking both gradient fields into account conjointly in non-orthogonal grid environments, when determining the vector of displacement in each gradient field, before performing vector addition, produces the exact goalward vector (purple solid arrow).

Grid map navigation appears to require both information regarding how gradients are distributed in space, allowing animals to structure their map, and an implementation strategy for the combination of multiple gradients to determine the direction of a goal. The complexity of both of these steps is likely to depend on how the gradients are distributed in space. For instance, navigating with non-linearly and non-orthogonally varying gradients may require complex mechanisms to solve exactly. Further, gradients that vary at close to parallel with each other have typically been assumed to be unsuitable for use in grid map navigation for various reasons (Gill & Taylor, 2024; Wynn et al., 2022), but particularly because of the magnifying effect on positional inaccuracy of any error in such environments (Bostrom et al., 2012; Åkesson & Alerstam, 1998). Conversely, if two gradients which vary linearly and orthogonally are used to navigate, determining the distribution of these cues and how to combine them may be simpler. Accurate navigation could be achieved through computing a vector of displacement from the goal in each gradient field separately and performing vector addition to determine a goalward vector (Figure 1A). In non-orthogonal gradient fields, the same mechanism would produce predictable biases in orientation, as shown in Figure 1B. Nonetheless, Benhamou suggested that this might be a simple mechanism by which animals might approximately navigate in orthogonal and non-orthogonal grid environments (Benhamou, 2003), and this has been termed the approximate model of combining gradient fields (Turner et al., 2016)

This navigational model was suggested by Benhamou to be simpler for animals to execute than taking the gradient fields into account conjointly to produce an exact goalward vector in non-orthogonal grids (shown in Figure 1C). This has been expanded upon by Turner and colleagues, in a study in which they examine navigation in more complex gradient fields (e.g. curvilinear or exponentially-varying fields), and suggest a range of navigational models that animals might utilise (Turner et al., 2016). These models involve different strategies of combining multiple gradients. Turner and colleagues model three mechanisms by which animals could combine two gradient fields to produce goalward orientations: the approximate model (the mechanism modelled by Benhamou and introduced above), which involves determining the displacement in each gradient field independently before performing vector addition; the directional model, which uses only the direction with which the gradient fields vary, ignoring their magnitudes, and weighing the direction of displacement in each gradient field equally before averaging them to orient; and the correct model, which takes the fields into account conjointly when determining the vector of displacement in each gradient field, before performing vector addition to find the correct vector home with linear gradient fields. In simple grid environments, with gradient fields varying linearly and orthogonally, only the directional model produces orientation errors, with the other two models computing exact goalward vectors. With gradients varying linearly but non-orthogonally, both the directional and approximate model produce distinctive patterns of orientation error, with the correct model taking the two fields into account conjointly to determine the exactly accurate goalward vector.

Turner and colleagues assume that animals generate a map structure from the distribution of gradients through linear extrapolation. In simpler environments, with gradients varying linearly, linear extrapolation of the gradient fields does not generate any further errors in orientation, beyond those potentially generated through the mechanism of combining multiple gradient fields. Conversely, in environments with curvilinear and exponentially-varying gradients, linear extrapolation of the gradient fields to generate a map structure should produce orientation errors, with the spatial pattern of these errors depending on the mechanism of extrapolation. Turner and colleagues set out two models for extrapolating gradient fields: in the first model, the gradient fields are linearly extrapolated from the direction and magnitude of the fields at the target/goal location; in the second, the gradient fields are linearly extrapolated from the direction and magnitude of the fields at the ‘release’ locations (any locations from which they orient goalward). A third model that could be introduced might involve extrapolating with average vector along which the fields vary across the distribution of experienced locations. These three models, in conjunction with models for combining gradient fields (including the ‘correct’ model), produce distinct predictions of the spatial patterns of orientation errors in complex environments with curvilinear and exponentially-varying gradients.

One factor that has received little consideration is the ontogeny of grid map navigation mechanisms. Tinbergen emphasised ontogeny as one of the major problems or questions for the field of ethology, defining it as the change in behaviour machinery during development (Tinbergen, 1963). The mechanisms involving map structuring through extrapolating gradient fields and the strategies for combining multiple gradients to determine goalward vectors can be considered as some of the behaviour machinery involved in grid map navigation. One possibility is that animals might be predisposed to utilise particular map structures and strategies for combining multiple gradients, and so these mechanisms might be relatively invariant through development. In this case, if animals utilise some of the mechanisms of extrapolating and combining gradients that have been proposed (Benhamou, 2003; Turner et al., 2016), we might expect to find patterns of orientation errors in animal tracking that match up with the predictions of these models. Conversely, these mechanisms might arise and change during development through learning, and hence our expectations might be more nuanced: whether we would expect to find the signature error patterns of these proposed models, and at what point in development, is not immediately clear.

We sought to examine how mechanisms of grid map navigation might arise and change through learning by training artificial neural networks to compute goalward directions in simulated environments. We used artificial neural networks called multi-layer perceptrons which can act well as simple computational models of animal learning and have been shown to be capable of replicating various empirical features of animal learning (Ghirlanda & Enquist, 2007; Gluck, 1991; McClelland & Rumelhart, 1985; Peng & Chittka, 2017; Wisniewski et al., 2012). Neural networks were trained at a large number of locations within a simulated environment at using gradient information to output directions towards a single goal in the centre of the environment. At various stages of training, we tested whether the errors of the networks conformed to predicted error patterns under different models of grid map navigation. It is difficult to assess how analogous the training of artificial neural networks in this way is to navigational learning in animals, and caution must be applied to any findings to ensure that they reflect modelled biology as opposed to the specifics of network implementation (Dawson, 2008; Franks & Ruxton, 2010). We therefore used two different implementations in order to ensure that the results did not rely on the precise way in which the networks outputted the direction to the goal or were optimised through training. This method allowed us to examine the ways in which, at least in theory, grid map navigation mechanisms could change through development. This facilitates more nuanced predictions about how signature error patterns of navigational models might manifest through development and in adult animals. We hope that this might prove useful in the coming years as contemporary developments in animal tracking technology facilitate the proliferation of large datasets in which these predictions can plausibly be tested.

## Methods

### The artificial neural networks

We utilised two implementations to train artificial neural networks (multi-layer perceptrons) to compute goalward directions using simulated gradients, with at least hundred replicates in each simulated environment (see precise sample sizes below). In both implementations, neural networks had 2 input neurons, each representing the magnitude of one of the two gradient cues in the simulated environment, and 6 Dense hidden layers (1^st^: 10 neurons; 2^nd^: 100; 3^rd^: 200; 4^th^: 500; 5^th^: 200; 6^th^: 100) with a Rectified Linear Unit (ReLU) activation function (Chollet, 2015; Gulli & Pal, 2017). This function, now standard in deep learning, was initially proposed in early theoretical models of nerve activation (Householder, 1941). In **implementation 1**, we treated the task as a regression problem; the neural networks had a single output neuron constrained between 0 and 1 with a sigmoid activation function. This output represented the predicted direction of the goal, with units of degrees/360. To control for any biases generated by the implemented baseline direction of the output (an output of 0 degrees/360), in each replicate, a baseline direction was randomly assigned to each neural network, such that an output of 0 degrees/360 corresponded to a random direction. The loss function, which is minimised during training, was the squared directional error of each neural network prediction. Standard gradient descent was used to optimise the networks during training (learning rate = 0.001; Nesterov momentum = 0.9). In **implementation 2**, we treated the task as a categoric problem; the neural networks had 20 output neurons, with sigmoid activation functions, each representing an equally sized sector of the circle, with the neural networks estimating which of the sectors encompassed the goal direction. Like in the first implementation, to avoid biases generated by the implementation of the directions represented by the set of 20 sectors, and to improve the coverage of the results, the directions represented by the set of sectors was randomly assigned to each neural network. To optimise classification of goalward directional sectors, a binary crossentropy loss function in combination with the Adam optimiser was used in this implementation. In both cases, network training took place over a single epoch and with a batch size of one; hence networks were effectively trained with information relating to a single spatial location at a time and each location only once, in an effort to constrain learning in a biologically realistic way. This neural networks were implemented in Python 3.10.7 (Van Rossum & Drake Jr, 1995), with the libraries: keras (Chollet, 2015), sys, numpy (Harris et al., 2020), scipy (Virtanen et al., 2020), matplotlib (Hunter, 2007) and tensorflow (Abadi et al., 2016).

### The simulated environments

**Figure 2:**
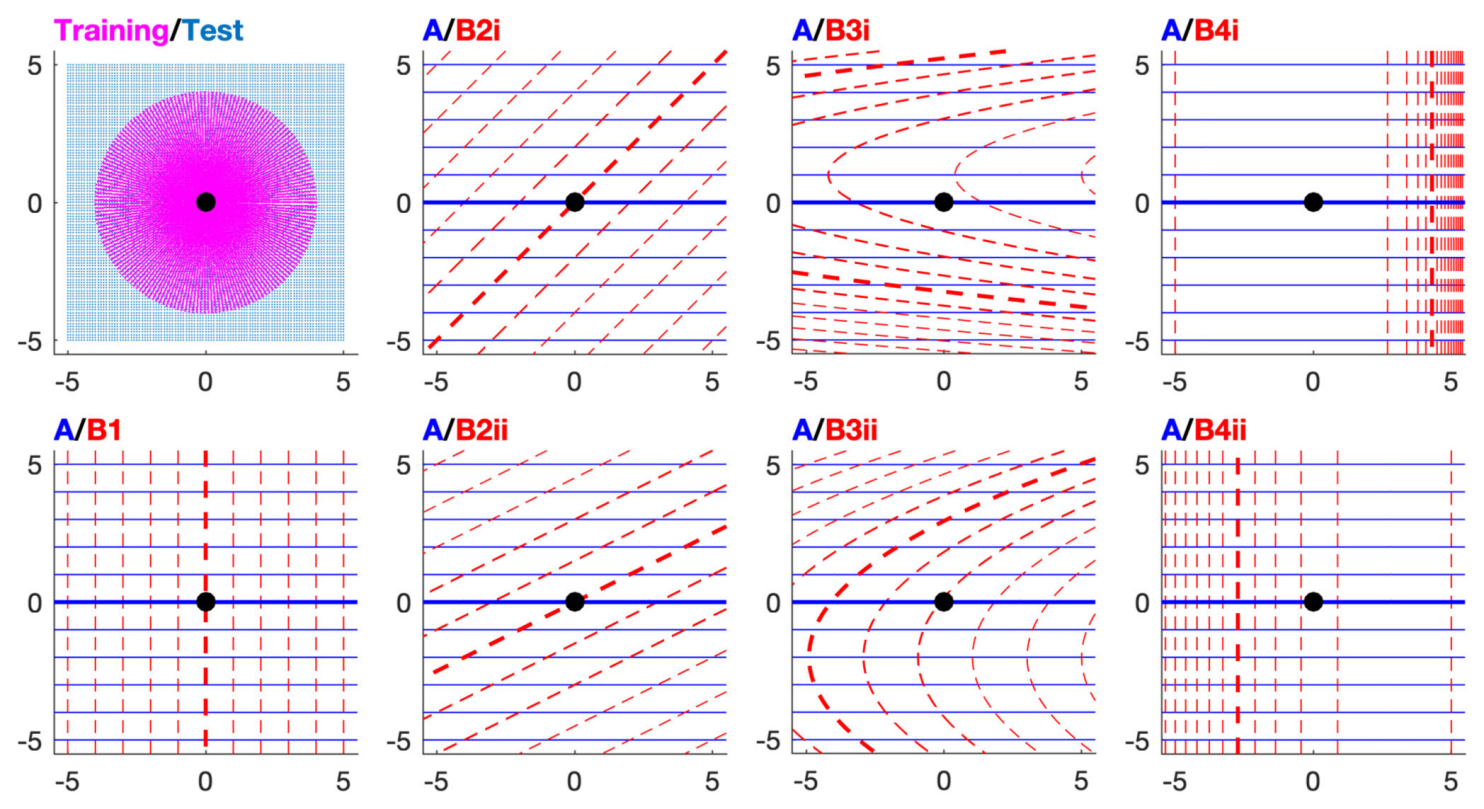
The simulated environments. The top left panel shows the locations used to train (in magenta) and test (in blue) the neural networks within the simulated environments. The other seven panels show the seven simulated environments in which neural networks were trained and tested, each comprising two gradients. Gradient A is the same in every environment and is shown with solid blue contour lines; gradient B is different in each environment and is shown with dashed red contours. The contour lines are spaced one unit apart in gradient value; where the gradient value is equal to zero, a thicker contour line is used. In every environment, the goal location is the same, at the origin of the Cartesian coordinate system, and represented by a black point in each panel.

The artificial neural networks were trained and tested to output goalward directions in a range of simulated environments. In all environments, the target of the neural networks was set at (0, 0) in the Cartesian coordinate system. Possible training locations were generated through all combinations of sets of distances and directions from the target, with the set of distances spaced 0.025 units apart between 0.025 and 4 units from the target, and the set of directions spaced 4 degrees apart, from the y axis all the way round the circle. This procedure generated a greater density of training locations closer to the target. Testing locations were regularly spaced every 0.1 units along both the Cartesian x and y axes from -5 to 5 in both axes. The training and test locations are shown in the top left panel of Figure 2.

Every simulated environment comprised two gradient fields. Seven different environments were simulated, conforming to the first four general cases modelled by Turner and colleagues (their 5^th^ case is excluded, which examines gradient fields containing anomalies). In all cases, one field (A) varied linearly. The second gradient field varied either: (B1) linearly and orthogonally to field A; (B2i and B2ii) linearly and non-orthogonally to field A; (B3i and B3ii) non-linearly and non-orthogonally to field A at the target; (B4i and B4ii) non-linearly and orthogonally to field A. The equations of gradient A and gradient B in each of the simulated environments are given in Table 1, where the *x* and *y* represent the axes of a Cartesian coordinate system, and each *k* represents a constant value determined such that all of the gradients varied over the same range within the simulated environment. The seven simulated environments are shown in Figure 2. The simulated environments will be referred to in relation to their B gradient henceforward (e.g., environment B2i).

### Generating predictions with navigational models

We use and extend the navigational models set out by Benhamou and by Turner and colleagues in previous modelling studies (Benhamou, 2003; Turner et al., 2016). See Turner et al, 2016 for mathematical descriptions of these models. The neural networks were tested in how closely their testing outputs conformed to each of the navigational models. The models include three ways in which two linear gradient fields are combined to generate goalward directions:

1) **The Approximate Model (APPROX)**. This comprises computing a vector of displacement from the goal in each gradient field independently and then determining an overall vector of displacement through vector addition (see Figure 1).
2) **The Directional Model (DIRECTIONAL)**. This model ignores the magnitudes with which the gradient fields vary, using only the directions along which they vary. The model comprises computing the direction of displacement from the goal location in each gradient field, and then weighing the two directions equally, irrespective of the magnitude of the displacement in each of the gradient fields.
3) **The Correct Model (CORRECT)**. This model correctly takes two linear fields into account conjointly to compute a vector of displacement in each gradient field and then determining an overall vector of displacement through vector addition. This produces the correct goalward direction, assuming both fields vary linearly.

**Figure 3:**
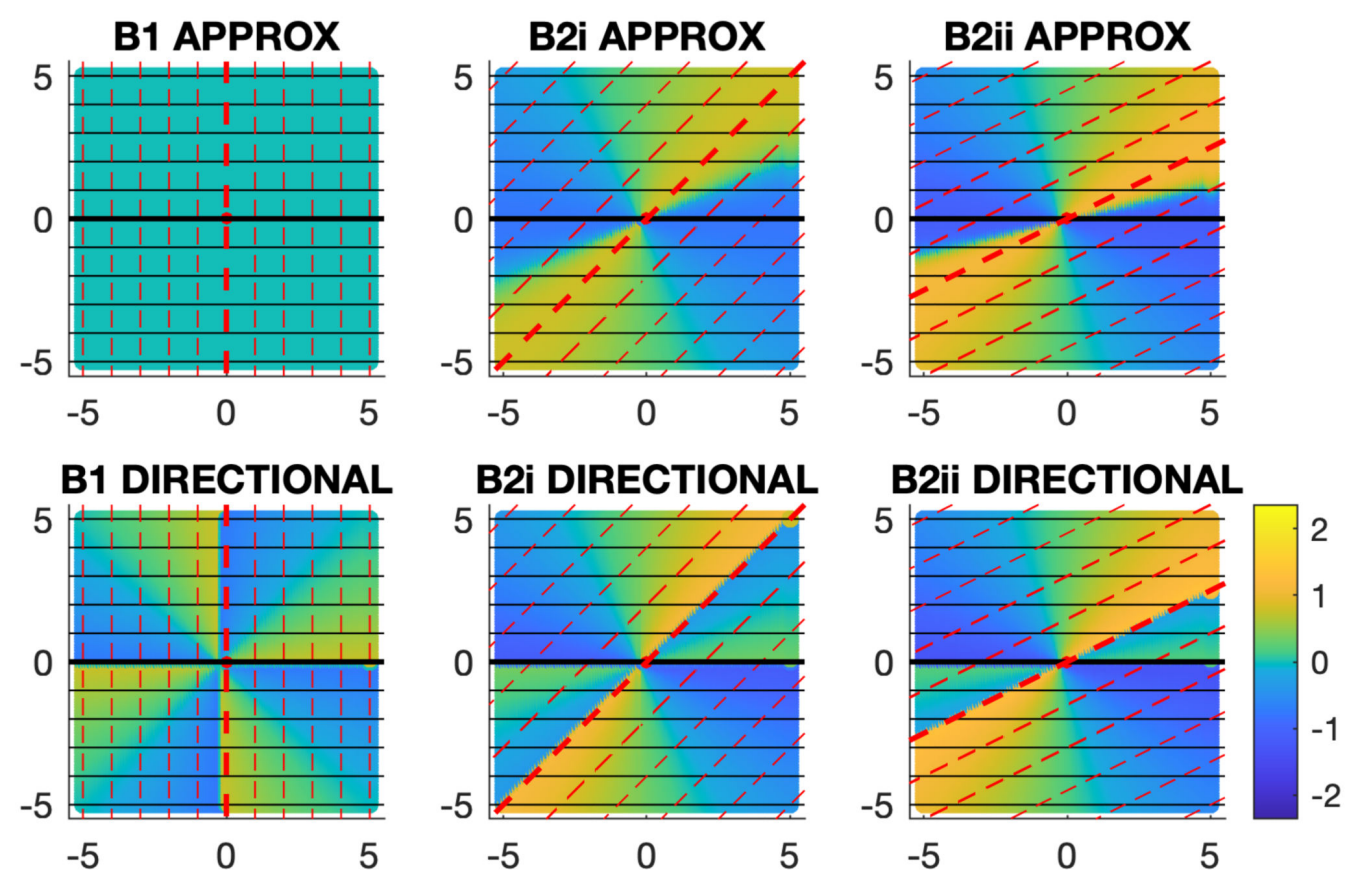
Model predictions in simpler environments. The orientation errors for the testing locations predicted by the approximate and directional models in orthogonal (B1) and non-orthogonal (B2i and B2ii) linear environments are shown by the heatmaps. The angular error scale in radians is shown bottom-right, with anticlockwise error positive (brighter colours) and clockwise error negative (darker colours). The gradients are represented as in Figure 2.

In simpler environments, with linear gradient fields, the correct model produces accurate orientation predictions, whereas the approximate model produces orientation errors in non-orthogonal environments and the directional model produces orientation errors in all environments. The orientation errors produced by the approximate model and the directional model in simulated environments with gradient A and either gradient B1, B2i or B2ii are shown in Figure 3.

In more complex environments with non-linear gradient fields, these models are extended to include linear extrapolations of the non-linear gradient fields. Three mechanisms of linear extrapolation are modelled, extending the models of Turner and colleagues:

1) **Target-based extrapolation (T)**. The gradients are linearly extrapolated from the target/goal location with the direction and magnitude of the fields at the target site.
2) **Release-based extrapolation (R)**. The gradients are linearly extrapolated from each release (training or test) location, with the direction and magnitude of the fields at each of those sites.
3) **Training-based extrapolation (Tr)**. The gradients are linearly extrapolated using the average vector along which the fields vary across the distribution of training locations.

**Figure 4:**
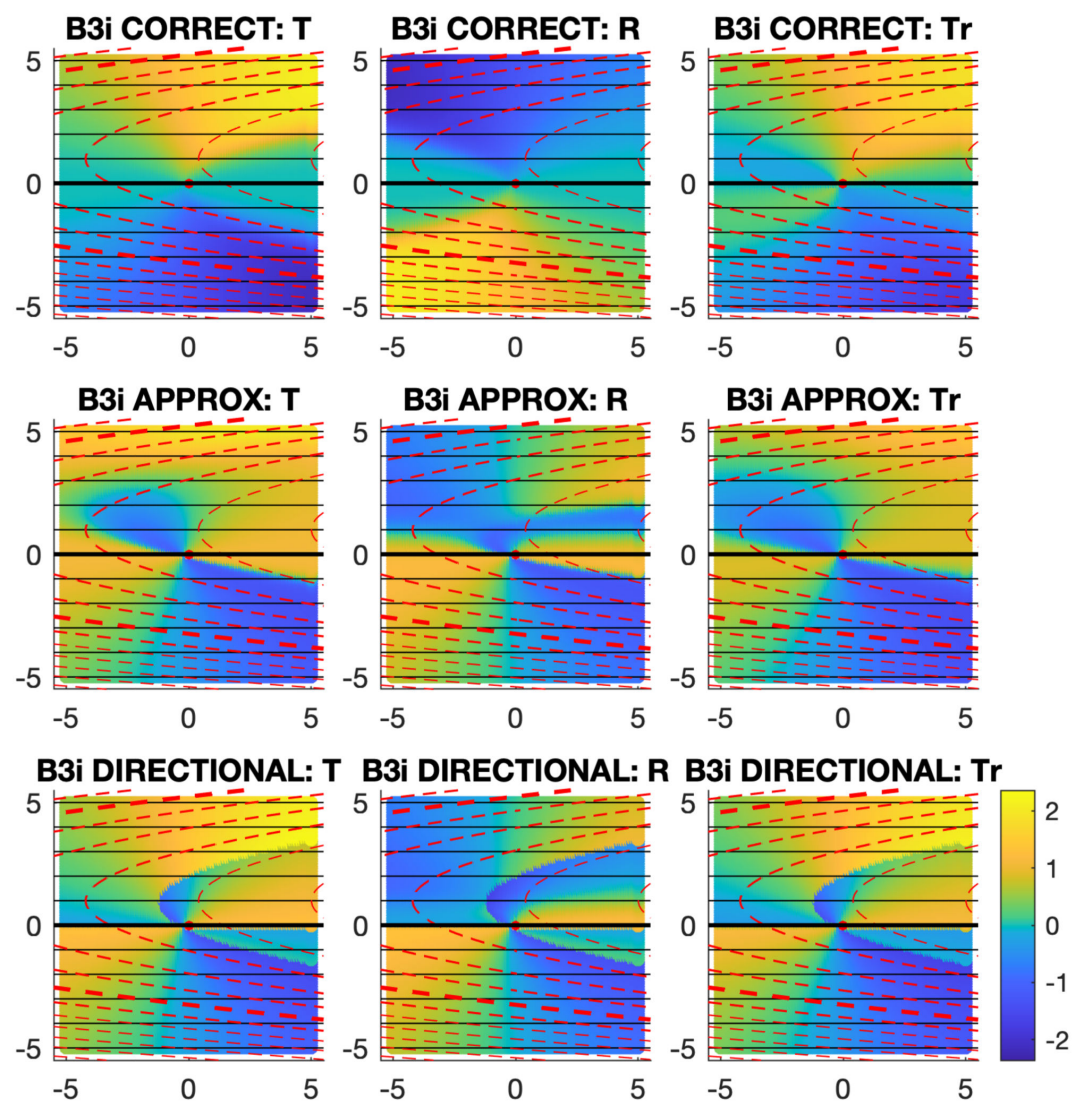
Model predictions in a non-linear, non-orthogonal environment. The predicted orientation errors at the testing locations of the nine different navigational models in the simulated environment with gradients A and B3i are shown. The angular error scale in radians is shown bottom-right, with anticlockwise error positive (brighter colours) and clockwise error negative (darker colours). The gradients are represented as in Figure 2.

These three models of combining gradient fields in combination with three models of linearly extrapolating gradient fields produce 9 navigational models with different orientation error predictions in complex environments, with non-linear non-orthogonal gradients such as in the simulated environment with gradients A and B3i, as shown in Figure 4.

**Figure 5:**
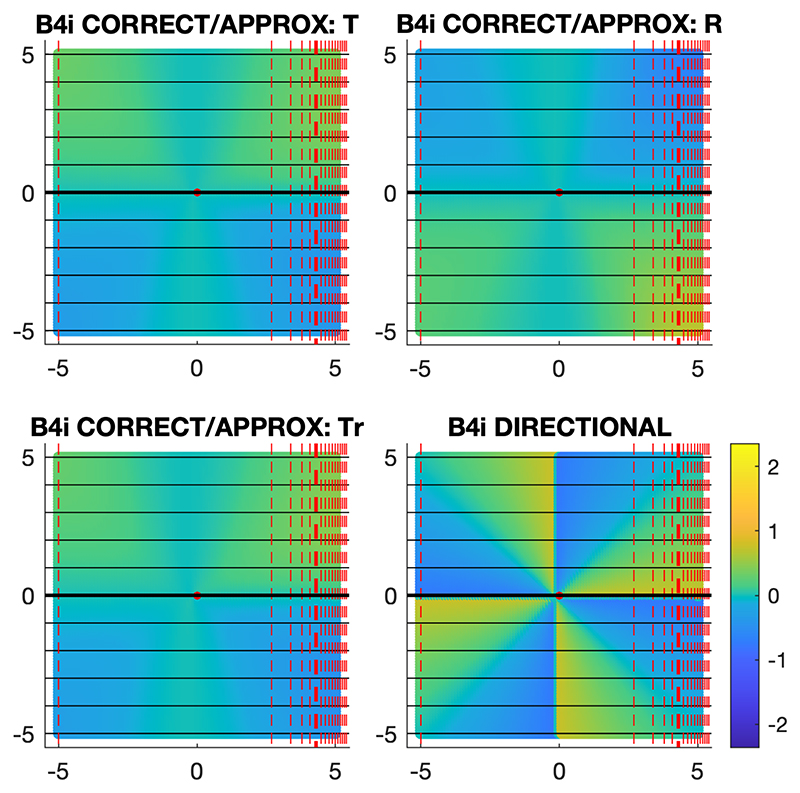
Model predictions in a non-linear, orthogonal environment. The predicted orientation errors at the testing locations of different navigational models in the simulated environment with gradients A and B4i are shown. The angular error scale in radians is shown bottom-right, with anticlockwise error positive (brighter colours) and clockwise error negative (darker colours). The gradients are represented as in Figure 2. The correct and approximate models make identical predictions in this environment, and there is no predictive distinction between directional models with different linear extrapolations of the gradients.

In certain environments, some of the models become equivalent, meaning that not all of the models can necessarily be distinguished. For instance, in environments with gradients varying non-linearly but orthogonally, the correct and approximate models make identical predictions of orientation error, and the different models of extrapolation make identical predictions when combined with the directional model of combining gradients. This is the case in the simulated environment with gradients A and B4i, as shown in Figure 5.

### Testing neural network performance against model predictions

We trained 100 artificial neural networks in each of the simulated environments (and under each of the two neural network implementations), except for environments B3i and B3ii, for which there were a greater number of navigational model predictions to distinguish, and so 200 replicates were used. Artificial neural networks were tested at various stages of training: after 0, 100, 250, 500, 1000, 2000, 4000, 8000 and 14000 training datapoints. The neural networks were tested at the testing locations shown in the blue points in Figure 2A. Neural network performance was tested against perfectly accurate performance and against every navigational model with unique, non-zero predictions of orientation error. The absolute angular error of the neural network output was calculated at every testing location for each artificial neural network, and the mean was taken across the testing locations, to produce a mean error for each neural network. The absolute angular difference between model predictions and the neural network output was calculated at every testing location for each artificial neural network, and the mean was taken across the testing locations, to produce a mean absolute difference for each neural network to a given navigational model’s predictions. This was computed for each of the navigational models. The fit of the different navigational models to the performance of the population of neural networks was then assessed using linear mixed effect models, of the general formula:

MeanAbsoluteDifference~NavigationalModel+(1|NeuralNetworkID)

Navigational Model was a categoric explanatory variable. Neural Network ID (assigned uniquely to each neural network replicate) was modelled with a random intercept. At each stage of training, the best-fitting model (with the lowest mean difference to the neural network outputs) is reported, along with significance at 5% significance level of a t-test (using Satterthwaite’s method (Kuznetsova et al., 2017; Satterthwaite, 1946)) to compare the fit between the best fitting model and the next best fitting model. Further, we report the significance at 5% significance level of a t-test to compare the fit of the neural network outputs to the best fitting model as compared to their fit to perfectly accurate performance (included in the statistical analysis as an additional navigational model), which was used as a baseline to assess model performance. Statistical analysis and graphical output was produced using Matlab (The-MathWorks-Inc., 2021)and R (R-Core-Team, 2018; RStudio-Team, 2020), using the R packages: circular (Agostinelli & Lund, 2022), plyr (Wickham, 2011), ggplot2 (Wickham, 2016), lme4 (Bates et al., 2015), vctrs (Wickham et al., 2023) and lmerTest (Kuznetsova et al., 2017).

## Results

Under each of the two neural network implementations, we trained 100 artificial neural networks in each of the simulated environments, except for environments B3i and B3ii, in which 200 were trained. As expected, under both implementations and in each simulated environment, neural networks began with an average absolute error of approximately half pi radians with no training, before improving through training. Neural networks achieved better performance most quickly in the simplest simulated environment with linear and orthogonal gradient fields (B1), and more slowly in the most complex environments, particularly B3i and B4i.

### Linear, orthogonal environments

**Figure 6:**
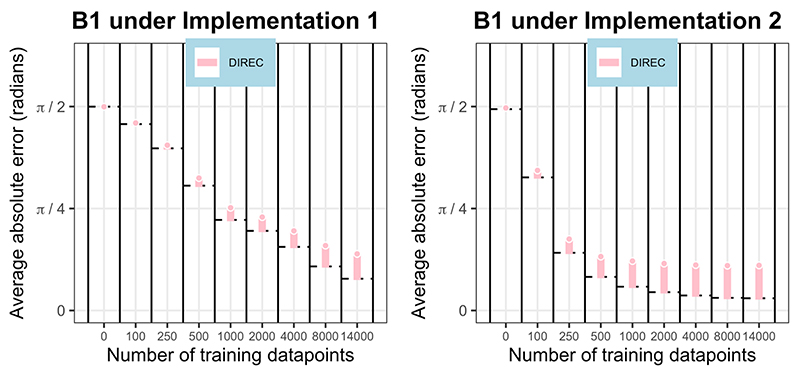
Model fit in linear, orthogonal environments. The orientation differences (average absolute angular error) between the neural network outputs and the predictions of the directional model are shown at various stages of training. These differences are presented relative to the orientation error of the neural networks from perfectly accurate performance, which acts as a baseline, and is represented as a black dotted line at the base of the bars at each stage of training. Values of orientation error below the baseline indicate a better fit between the neural network outputs and model predictions than between neural network outputs and perfectly accurate orientation performance. Combination model (DIREC=directional) legends are shown. The x axis is not to scale. Results from neural networks under implementation 1 are shown on the left, and those under implementation 2 on the right.

Only one navigational model, the directional model, predicts deviations in orientation from perfectly accurate performance in the B1 environment comprising linear, orthogonal gradients. The orientation differences (average absolute angular error) between the neural network outputs and the predictions of the directional model, relative to the orientation error of the neural networks from perfectly accurate performance (used as a baseline), are presented in Figure 6. At all stages of training, the neural networks conformed better (with lower average absolute error) to perfectly accurate performance than the directional model’s predictions.

### Linear, non-orthogonal environments

**Figure 7:**
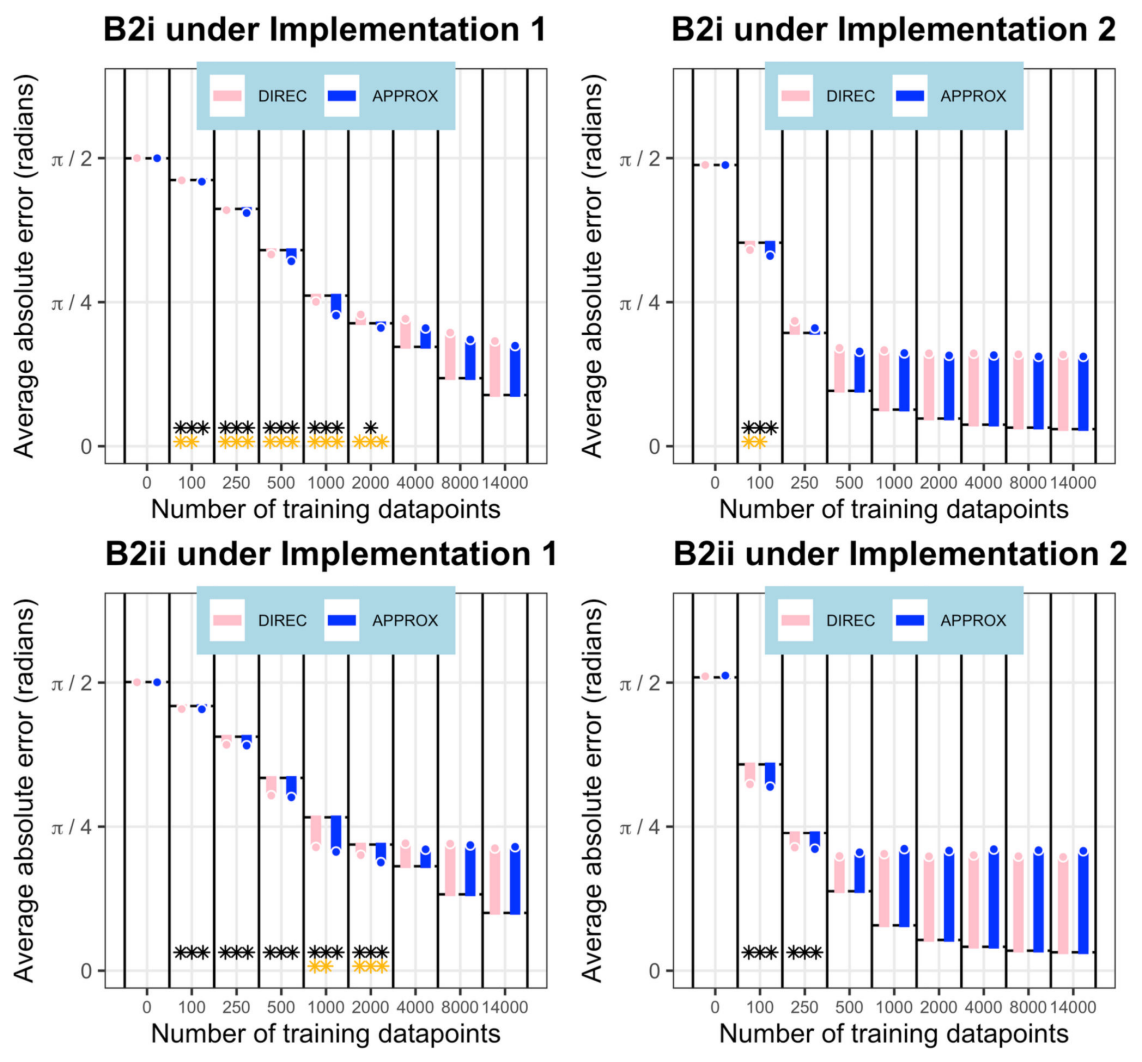
Model fit in linear, non-orthogonal environments. The orientation differences (average absolute angular error) between the neural network outputs and the predictions of both the approximate and directional models are shown at various stages of training. These differences are presented relative to the orientation error of the neural networks from perfectly accurate performance, which acts as a baseline, and is represented as a black dotted line at the base of the bars at each stage of training. Values of orientation error below the baseline indicate a better fit between the neural network outputs and model predictions than between neural network outputs and perfectly accurate orientation performance. Combination model (APPROX=approximate; DIREC=directional) legends are shown. The x axis is not to scale. Results from neural networks under implementation 1 are shown on the left, and those under implementation 2 on the right. Black stars show where the neural network predictions are significantly closer to the best fitting model’s predictions than to the baseline of the perfectly accurate solution (number of stars denotes significance level). Gold stars show where the best fitting model (with a better fit than the baseline) is a significantly better fit than the next best fitting model (number of stars denotes significance level).

Likewise, 100 neural networks under each implementation were trained in two environments with linearly but non-orthogonally varying gradients, environments B2i and B2ii. In these environments, both the directional and approximate models predict deviations in orientation from perfectly accurate performance. In both environments and under both implementations, after some training, the **approximate model** fitted the neural network outputs best, and significantly better than their fit to the perfectly accurate solution (p<0.001 at some stage of training in each case), as shown in Figure 7. In each of these cases, the approximate model also fitted significantly better than the directional model at some stage of training (p<0.01 at some stage of training in each case), except for the B2ii environment under implementation 2 (no significant difference between the approximate and directional model; p>0.05). By the end of training, in all of these cases, the neural network outputs conformed better to the baseline of perfectly accurate performance than the predictions of the approximate model.

**Figure 8:**
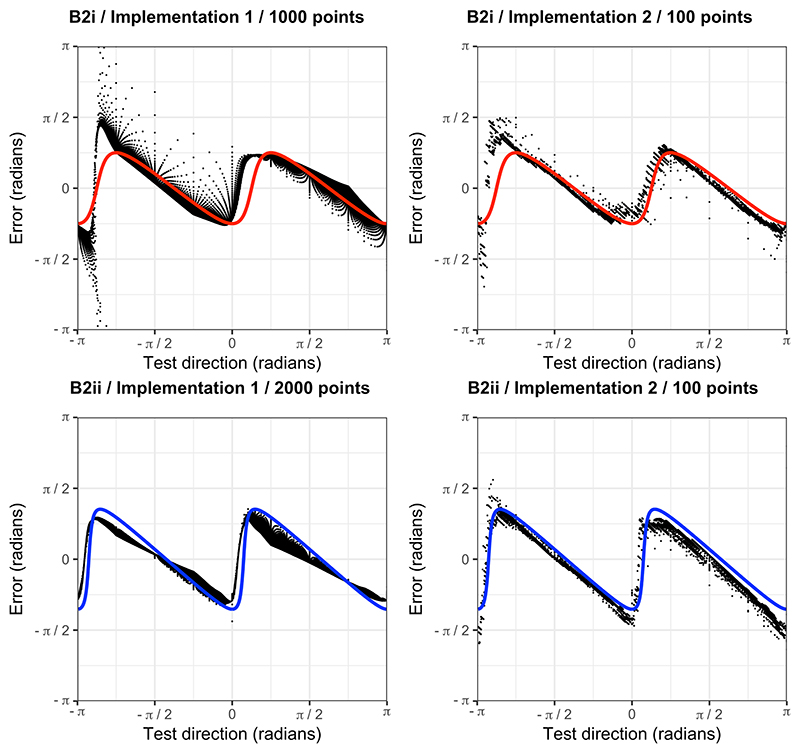
Neural network error and the approximate model predictions. From each neural network implementation, in each of environments B2i and B2ii, the performance of neural networks is shown at the stage of training at which the approximate model is the best fitting model by the largest margin. Each black point represents the average (signed) error of the neural networks at a single testing location. The predicted error under the approximate model at different directions from the target is shown by the red (B2i) and blue (B2ii) lines.

Figure 8 shows the predicted (signed) orientation errors of the approximate model at testing locations in B2i and B2ii across the range of possible directions from the target, and the average error of the neural networks across these directions, at the stage of training at which the approximate model was the best fitting model by the largest margin. Visual inspection reveals a very close fit of the model predictions to the average neural network outputs.

### Non-linear, non-orthogonal environments

**Figure 9:**
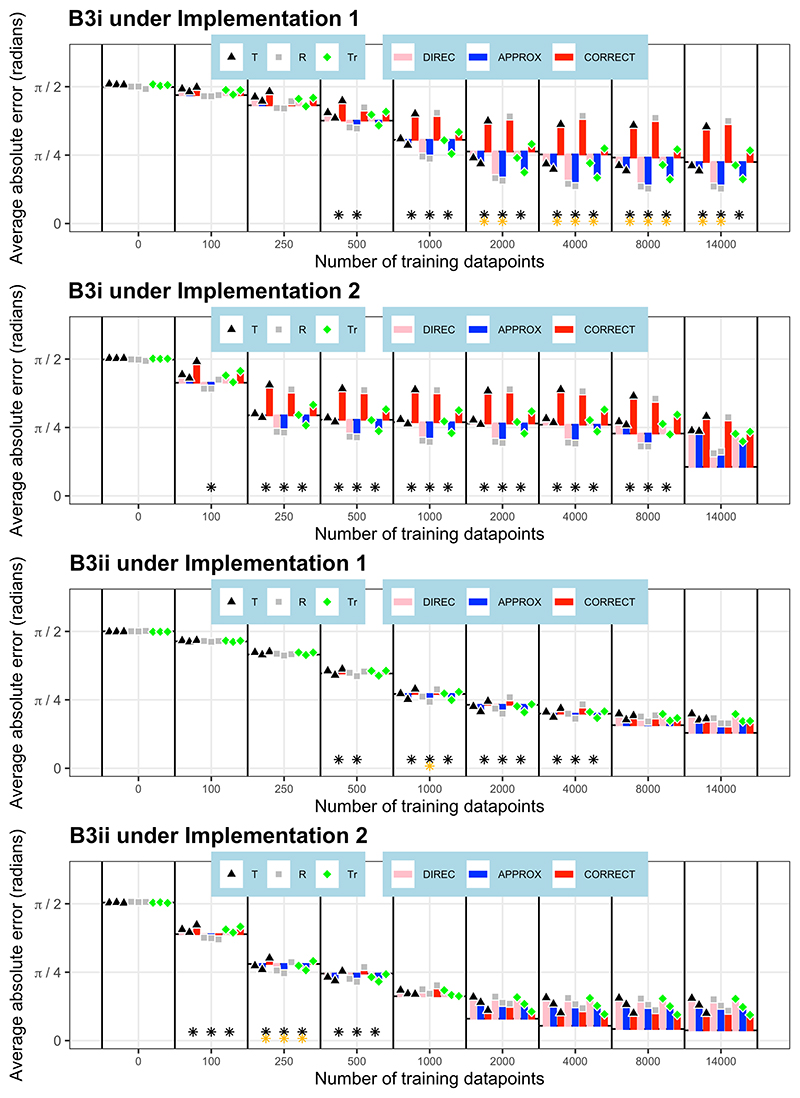
Model fit in non-linear, non-orthogonal environments. The orientation differences (average absolute angular error) between the neural network outputs and the model predictions are shown at various stages of training. These differences are presented relative to the orientation error of the neural networks from perfectly accurate performance, which acts as a baseline, and is represented as a black dotted line at the base of the bars at each stage of training. Values of orientation error below the baseline indicate a better fit between the neural network outputs and model predictions than between neural network outputs and perfectly accurate orientation performance. Combination model (APPROX=approximate; DIREC=directional; CORRECT=correct) and extrapolation model (T=target-based; R=release-based; Tr=training-based) legends are shown under the figures. The x axis is not to scale. Results from neural networks under implementation 1 are shown on the left, and those under implementation 2 on the right. At each stage of training, black stars show if the neural network predictions are significantly closer to the best fitting model’s predictions than to the baseline of the perfectly accurate solution (number of stars denotes significance level). Gold stars show if the best fitting model (with a better fit than the baseline) is a significantly better fit than the next best fitting model (number of stars denotes significance level).

Under more complex simulated environments, there are a greater number of navigational models that predict deviations in orientation from perfectly accurate performance, comprising two separate components. These involve both a model for combining the two gradients (directional, approximate, and correct models) and a model for extrapolating the gradients (target-based, release-based and training-based extrapolations). In the environments with gradients varying non-linearly and non-orthogonally at the target site, B3i and B3ii, 200 neural networks were trained under each of the two neural network implementations. In environment B3i, under both implementations, the **approximate model with release-based extrapolation** was the best-fitting model after some training, fitting the neural network outputs significantly better than perfectly accurate performance (p<0.001 at some stage of training in each case). Under the continuous output implementation (implementation 1), this model significantly outperformed all other models in later training (p<0.001), whereas under implementation 2, this model significantly outperformed all other models except for the directional model with release-based extrapolation (p>0.05). At the end of training, under implementation 1, the approximate model with release-based extrapolation was still the best-fitting model, whereas under implementation 2, the neural network outputs better conformed to the perfectly accurate solution than any of the model predictions. Similarly in environment B3ii, under both implementations, the **approximate model with release-based extrapolation** was the best-fitting model at some stage of training, fitting the neural network outputs significantly better than perfectly accurate performance (p<0.001 at some stage of training in each case) and all other models (p<0.05 at some stage of training in each case). There was a single exception: after 100 training datapoints under implementation 2, the correct model with release-based extrapolation was the best-fitting model, outperforming the approximate model with release-based extrapolation, but not significantly so (p>0.05). At the end of training, the neural network outputs better conformed to the perfectly accurate solution than any of the model predictions. These results are shown in Figure 9.

### Non-linear, orthogonal environments

**Figure 10:**
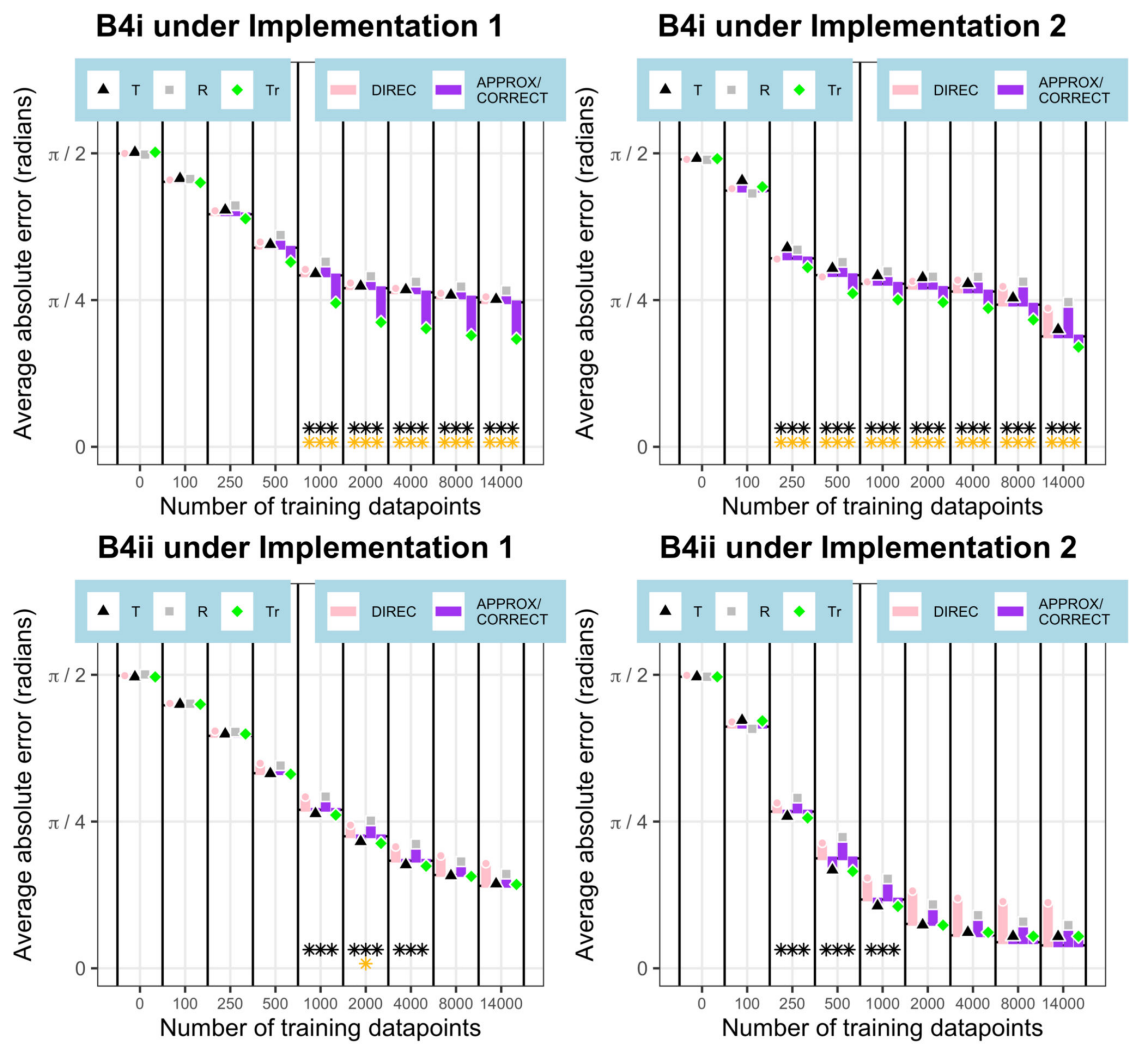
Model fit in non-linear, orthogonal environments. The orientation differences (average absolute angular error) between the neural network outputs and the model predictions are shown at various stages of training. These differences are presented relative to the orientation error of the neural networks from perfectly accurate performance, which acts as a baseline, and is represented as a black dotted line at the base of the bars at each stage of training. Values of orientation error below the baseline indicate a better fit between the neural network outputs and model predictions than between neural network outputs and perfectly accurate orientation performance. Combination model (APPROX/CORRECT=approximate / correct model; DIREC=directional) and extrapolation model (T=target-based; R=release-based; Tr=training-based) legends are shown under the figures. The x axis is not to scale. Results from neural networks under implementation 1 are shown on the left, and those under implementation 2 on the right. At each stage of training, black stars show if the neural network predictions are significantly closer to the best fitting model’s predictions than to the baseline of the perfectly accurate solution (number of stars denotes significance level). Gold stars show if the best fitting model (with a better fit than the baseline) is a significantly better fit than the next best fitting model (number of stars denotes significance level).

Finally, we trained 100 neural networks under each implementation in environments with non-linearly and orthogonally varying gradients, B4i and B4ii. In these environments, the approximate and correct models produce the same predictions for orientation error and so become the approximate/correct model. Here, in every case, **the approximate/correct model with training-based extrapolation** was the best-fitting model at some stage of training, with neural network outputs significantly better conforming to this model than to the perfectly accurate solution (p<0.001 at some stage of training in each case); this is shown in Figure 9. In three out of the four cases, this model significantly outperformed all others after some training (p<0.05 at some stage of training in each case). The exception was the B4ii environment under implementation 2, in which this model outperformed all others except for the approximate/correct model with target-based extrapolation (p>0.05). At the end of training, in the B4ii environment, the neural network outputs under both implementations better conformed to the perfectly accurate solution than to any of the navigational models, whereas in the B4i environment, the approximate/correct model with training-based extrapolation was still a better fit to the neural network outputs at the end of training. These results are shown in Figure 10.

## Discussion

In general, we found that artificial neural networks were able to utilise the simulated environmental gradients to improve their navigational performance after some training, with the rate of improvement determined by both the complexity of the environment, and the type of neural network implementation. In all cases, by the end of training, average absolute error was below π/4 radians, and in many cases, this level of performance was far exceeded. These levels of performance would facilitate reasonably efficient progress towards goals and is similar to the levels of performance that might be expected in the initial orientations of animals towards far-off goals (Wallraff, 2001).

Our results indicate that the artificial neural networks adopted the approximate model suggested for animal navigation by Benhamou, in a range of environments, after some training (Benhamou, 2003). This model involves computing a vector of displacement from the goal in each gradient field independently and determining an overall vector of displacement through vector summation. It produces predictable orientation errors in non-orthogonal gradient fields as shown in Figure 1. Across non-orthogonal and linear environments (B2i and B2ii), non-linear and non-orthogonal environments (B3i and B3ii), and non-linear and orthogonal environments (B4i and B4ii, in which it cannot be distinguished from the correct model), the approximate model (combined with a model of extrapolation in non-linear environments) fitted the predictions of the artificial neural networks best of all the navigational models and significantly better than the perfectly accurate navigational solution at some stage of training. In some cases, as detailed above, the approximate model also significantly outperformed all other navigational models. Additionally, Figure 7 allows visual inspection of the close fit between the error predictions of the approximate model across the range of directions from the target and the signed errors at each training datapoint averaged across the population of neural networks at intermediate stages of training in B2i and B2ii. This is despite the much poorer fit of the predictions of the model to the outputs of random neural networks within the population.

This demonstrates how the signature of this navigational mechanism can emerge clearly at the level of the population despite being more difficult to detect in the outputs of individual neural networks because of individual-level biases.

However, in most cases, by the end of training (after 14000 training datapoints), the perfectly accurate navigational solution fitted the artificial neural network predictions better than the approximate model and all other navigational models. The only exceptions were the neural networks under implementation 1 trained in environments B3i and B4i, and the neural networks under implementation 2 trained in environment B4i. These two environments were particularly complex (Figure 2) and the neural networks learned to navigate in them more slowly than in the other environments. We expect that, given sufficient training, these results may have conformed to the patterns observed in the other scenarios, with the perfectly accurate navigational solution eventually fitting the artificial neural networks better than any of the navigational models. In sum, these results appear to indicate that after initial training the neural networks adopt the approximate model to perform grid map navigation, but that after more training, the neural networks improve upon this solution and converge on the perfectly accurate solution. This may lend support to the idea that the approximate model is simpler to execute than the perfectly accurate solution (Benhamou, 2003), and hence is adopted by the artificial neural networks first, but that the neural networks improve upon the approximate model solution later in training.

In more complex environments with non-linear gradient fields, there are a range of possible mechanisms that could be employed to navigate. The models that we tested here assumed a linear extrapolation of the gradient fields following Turner and colleagues (Turner et al., 2016), in combination with a mechanism of combining the two gradients. We tested three models of linearly extrapolating the gradient fields: extrapolating about the target, about each ‘release site’ (the training and test locations), and extrapolating with the average vector along which the gradients vary across training sites. However, various mechanisms that do not involve a linear extrapolation of the field are possible, and hence the mechanisms modelled here are a small set of the potentially available mechanisms by which navigation could take place in these complex environments. Nonetheless, the consistent finding that the approximate model, in combination with a mechanism of linearly extrapolating the gradient fields, fitted the neural network outputs best at some stage of training provides reasonably strong evidence that this model captures the mechanisms adopted by the neural networks across a wide range of environments. In environments with non-linear and non-orthogonally varying fields (B3i and B3ii), we found evidence for the neural networks adopting release-based extrapolation, whereas, with non-linear and orthogonally varying fields (B4i and B4ii), we found evidence for the networks adopting training-based extrapolation. The reasons for this difference are unclear but could stem from the fact that in the former environments, both the direction and magnitude of the fields vary in space, whereas in the latter environments, the directions in which the fields vary remain constant through space.

These results held across both implementations of neural networks, in which neural networks either outputted direction via a single continuous output neuron (implementation 1) or categorised direction into one of twenty segments with categoric output neurons (implementation 2). This shows that these results were not entirely contingent on the specifics of the neural network implementations, and hence may have captured something more general about how learning of these modelled navigational tasks might occur.

Nonetheless, it is important to consider how the general implementation of these models may have influenced the observed results, and how this might relate to animal navigation. The study design was inspired by movement patterns in during central place foraging and similar navigational behaviours, such as those seen in homing pigeons, where an animal repeatedly returns to a fixed goal from various locations. This contrasts with other navigational contexts, such as course correction following displacement during migration (e.g., Chernetsov et al, 2017), where similar mechanisms might be employed. However, we did not model any specific pattern of spatial exploration. Instead, the neural networks were trained on randomly ordered datapoints sampled from a distribution of locations with greater density near the target. While this distribution may approximate a realistic spatial structure during exploration, the random ordering of training datapoints likely diverges from the way animals typically acquire information through exploration. This difference may have contributed to the rapid performance improvements observed in our models. Further work could explore how different exploration patterns influence learning outcomes and the navigational strategies that emerge. Additionally, our models assumed that animals can perfectly detect the magnitude environmental gradients, that these gradients remain stable over the timeframe of learning, and that external factors such as wind, currents, or biased compasses exert no influence. Relaxing these assumptions would likely alter the rate of learning and affect the distribution of navigational errors (Painter & Hillen, 2015; Schneider et al., 2023; Wynn et al., 2022), potentially making characteristic error patterns harder to detect. However, it is unclear whether these sources of error would impact the navigational strategies adopted by animals; this remains an open question that could be addressed in further work using this modelling framework.

These findings have potential implications regarding the predictions of these navigational models for animal navigation. First, they lend support to the suggestion that the approximate model is simpler to execute than a perfect navigational solution in non-orthogonal environments, and might therefore be adopted by animals (Benhamou, 2003; Turner et al., 2016). Second, while it is possible that animals have fixed mechanisms for extrapolating and combining gradient fields such that the predictions of these models might be met consistently through development, these results demonstrate that if animals learn mechanisms to combine gradient fields in order to navigate, the predictions of these models might only be met in certain stages of development. These mechanisms might then be adopted at some stage of learning and not necessarily maintained through development, but instead improved upon through learning. This is an important qualifier to the predictions of these navigational models and could be crucial for the testing of these model predictions. However, the role of evolution, as well as learning, in tuning the mechanisms by which animals extrapolate and combine gradient cues has not been examined in our models and could predispose animals to navigate and learn to navigate in specific ways. Finally, these results show how navigational mechanisms adopted by animals could be difficult to detect at the individual level but could emerge clearly at the population level. We anticipate that the proliferation of large tracking datasets of animal movements might allow the predictions of these navigational models to be tested in the coming years. We suggest that full lifetime tracks of animal movements could be particularly valuable for testing for the adoption of these navigational models by animals and facilitate a better understanding of the precise mechanisms and ontogeny of long-distance navigational abilities in animals.

## Figures and Tables

**Table 1:** The gradient fields of the simulated environments. The equations defining the gradient fields of the 7 simulated environments are shown. Gradient A is the same in every simulated environment, whereas there are 7 versions of gradient B, conforming to 4 general cases. x and y represent the axes of a Cartesian coordinate system and each k represents a constant value, chosen to standardise the range of the gradient field in the simulated environment.

Gradient A:		
**All Cases**	*A* = *(x* – *k_1_A_)/k_2_A_*	
**Gradient B:**		
**Case 1**	*B1 = (y* – *k_1_B1_)/k_2_B1_*	
	**i**	**ii**
**Case 2**	*B2i = (x* – *y* – *k_1_B2i_)/k_2_B2i_*	*B2ii = (x* – *2y* – *k_1_B2ii_)/k_2_B2ii_*
**Case 3**	*B3i = (x* – *(y-1)^2^* – *k_1_B3i_)/k_2_B3i_*	*B3ii = (x* –*0.2(y + 2)^2^* – *k_1_B3ii_)/k_2_B3ii_*
**Case 4**	*B4i = (e^x^* – *k_1_B4i_)/k_2_B4i_*	*B4ii = ((6* –*x)^3^* – *k_1_B4ii_)/k_2_B4ii_*

## Highlights

- We trained artificial neural networks to navigate in virtual grid environments
- Neural networks adopted a mechanism termed the approximate model during training
- This supports the idea that animals might adopt this mechanism through learning
- Neural networks ultimately dropped this mechanism after further training
- Similarly, animals may adopt and alter navigational strategies as they learn
